# A Highly Magnetic Field Sensitive Photonic Crystal Fiber Based on Surface Plasmon Resonance

**DOI:** 10.3390/s20185193

**Published:** 2020-09-11

**Authors:** Huimin Huang, Zhenrong Zhang, Yang Yu, Lingjun Zhou, Yuyu Tao, Guofeng Li, Junbo Yang

**Affiliations:** 1 Guangxi Key Laboratory of Multimedia Communications and Network Technology, School of Computer, Electronics and Information, Guangxi University, Nanning 530004, China; 1813301006@st.gxu.edu.cn (H.H.); zzr76@gxu.edu.cn (Z.Z.); 1813391017@st.gxu.edu.cn (L.Z.); taoyuyu@st.gxu.edu.cn (Y.T.); 1813391006@st.gxu.edu.cn (G.L.); 2 Center of Material Science, College of Liberal Arts and Sciences, National University of Defense Technology, Changsha 410073, China; yangjunbo@nudt.edu.cn

**Keywords:** surface plasmon resonance, sensor, photonic crystal fiber, refractive index, magnetic field

## Abstract

A novel magnetic field sensor comprising a photonic crystal fiber (PCF) is designed and investigated based on surface plasmon resonance (SPR). We use finite element analysis in order to analyze the sensing characteristics of the magnetic field sensor. The simulation results show that the sensor is very sensitive to the change of refractive index and has good linearity in the refractive index range from 1.43–1.45. The thickness of the metal film and the metal material has great influence on the resonance wavelength and the peak of the loss spectrum, the diameter of the central air hole will affect SPP excitation. When the thickness of gold layer is 50 nm, the refractive index sensitivity is 4125 nm/RIU in the refractive index range from 1.43–1.45. Using the designed sensor for magnetic field sensing, the loss spectrum is red-shifted with the increase of the magnetic field, the highest magnetic field sensitivity can reach 61.25 pm/Oe in the range from 50 Oe to 130 Oe. The sensor not only has high sensitivity of refractive index, but it can also realize accurate measurement of magnetic field. It has huge application potential in complex environment, remote sensing, real-time monitoring, and other fields.

## 1. Introduction

Surface plasmon polariton (SPP) is a kind of surface electromagnetic wave propagating along the interface between metal and dielectric. SPP can enhance localized field and break the diffraction limit, possessing the ability to manipulate light in the subwavelength scale [1,2]. When SPP is generated and transmitted at the metal interface, it will form surface plasmon resonance (SPR). SPR is the oscillation of free electrons propagating along the metal-medium interface. In the early days, Kretschmann used a prism to irradiate light onto the gold-plated surface to excite SPR at the metal-analyte interface [3]. SPR has extremely high sensitivity to changes in the refractive index of the surrounding medium, so it is widely used in biosensing research fields, such as DNA detection [4,5]. However, the traditional Kretschmann prism device has a large structure and high cost, which is not suitable for remote sensing, thus limiting its large-scale manufacturing and application [6]; therefore, people have been looking for alternatives to Kretschmann prism devices. As optical fibers are widely used in sensors, optical fibers are considered to be an excellent platform for the development of miniaturized and low-cost SPR sensors. The core of the optical fiber is used as a prism. As long as a metal layer is plated around the core, the evanescent field of light can be used to excite SPR [7,8]. Optical fiber sensors that are based on surface plasmon resonance have a wide range of application prospects in the field of sensing, and they are increasingly favored by people. In the past years, many SPR-based optical fiber sensing structures have been proposed, such as D-shaped fiber type, tapered fiber type, TFBG type, photonic crystal fiber (PCF) type, etc. [9,10,11,12]. The condition for realizing SPR fiber sensing is to meet the phase matching condition, which is, the real part of the effective refractive index of the SPP mode and the waveguide mode are equal in value at a certain wavelength [13]. The structural design of PCF is flexible and optimized PCF structure parameters can achieve control of the mode field, so it is easier to achieve the phase matching of the fiber core mode and SPP mode [14]. When compared with other types of fiber optic SPR sensors, PCF-SPR sensors have the advantages of flexible structural design, high sensitivity, and stable mechanical structure, and they have received great attention. At present, scholars have continuously proposed new photonic crystal models to improve the sensitivity and sensing range of the designed PCF-SPR sensor by carefully designing the photonic crystal fiber structure and optimizing the fiber structure parameters [15,16]. In 2016, Liu et al. proposed a temperature sensor that was based on SPR. The structure includes a central air hole and six large liquid-filled holes. The central air hole can effectively reduce the effective refractive index of the fiber core mode, more easily meeting the phase matching condition. The sensor’s highest sensitivity can reach 3080 pm/°C [17]. In 2017, Zhu et al. proposed a photonic crystal fiber temperature and magnetic field dual-parameter sensor based on uniformly symmetrically arranged three-layer regular hexagonal pores. The temperature sensitivity of the sensor is up to −1.575 nm/°C and the magnetic field sensitivity is up to 4.333 μm/T [18]. The researchers have improved the sensitivity of the sensor and increased the function of the sensor by optimizing the structural parameters and using different filling liquids. However, the dynamic range they detect is not large. In the same year, a large detection range SPR sensor that is based on hollow-core photonic crystal fiber was proposed by Luan et al., the resonance properties between the core modes and the surface plasmon polariton modes excited in a large refractive index range from 1.33 to 1.5 [19]. In 2019, Kawsar Ahmed et al. designed the D-shaped elliptical dual-core photonic crystal fiber configuration with the compact sensing performances while infiltrating the blood fluid [20]. The research further expands the application range of PCF-SPR sensors. In 2020, Md. Nazmus Sakib et al. proposed a sensor with the optimized form of circular slotted lattice (CSL) structure. This model indicates the highest wavelength sensitivity of 16,000 nm/RIU and the average spectral sensitivity of 6666 nm/RIU [21]. This structure can be a strong competitor in the field of biosensing. However, the sensor requires more gold, which is costly, and the structure is difficult to manufacture. Therefore, a D-shape PCF temperature sensor that is based on SPR was designed by Md.Aslam Mollah et al. in 2020. The main motive is to attain high fabrication feasibility [22]. 

In response to the above problems, our proposed a PCF-SPR sensor that is simple to fabricate, the cost is low, the sensitivity is high, and it can meet the detection needs. In this paper, a new photonic crystal fiber magnetic field sensor based on SPR is proposed. We used COMSOL Multiphysics 5.4 based on finite element analysis to simulate the proposed sensor structure. The designed photonic crystal fiber has five layers of air holes. The air holes on both sides of the central air hole are missing, forming two cores A and B. The outer air holes effectively reduce the effective refractive index of the fiber core mode, making it easier to achieve phase matching. The central air hole is plated with metal on the inner wall, filled with magnetic fluid, and used as a sensing channel for exciting SPR. The dual-core PCFs and their mode coupling properties were widely investigated [23]. The dual-core design can effectively enhance the evanescent field, which makes it easier to stimulate SPR. The simulation results show that the sensor is very sensitive to the change of refractive index and has good linearity in the refractive index range from 1.43–1.45. The thickness and the diameter of the central air hole will affect the peak position and height and SPP excitation. When the thickness of gold layer is 50 nm, d_c_ = 2.8 μm, the refractive index sensitivity is 4125 nm/RIU, and the thickness of silver layer is 50 nm, d_c_ = 2.8 μm, the refractive index sensitivity is 4755 nm/RIU in refractive index range from 1.43–1.45. When the designed sensor is used for magnetic field sensing, the loss spectrum is red-shifted with the increase of the magnetic field. By optimizing the sensor structure parameters, the magnetic field sensitivity and resolution of the sensor can be improved. The highest magnetic field sensitivity can reach 61.25 pm/Oe in the range from 50 Oe to 130 Oe. The sensor not only has high sensitivity of refractive index, but can also realize accurate measurement of magnetic field. The sensor that we designed is easy to manufacture, and requires less metal and low production cost. It has huge application potential in complex environment, remote sensing, real-time monitoring, and other fields.

## 2. Model and Theory

**Figure 1:**
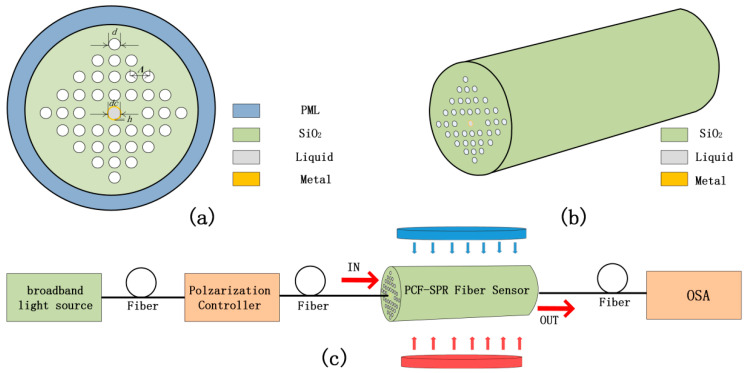
(**a**) The cross section of the proposed photonic crystal fiber-surface plasmon resonance (PCF-SPR) sensor; (**b**) Schematic diagram of the proposed PCF-SPR sensor in three-dimensional (3D) model; and, (**c**) Schematic diagram of the experimental set-up of the proposed sensor.

Figure 1a presents the schematic cross-section of the PCF-SPR magnetic field sensor. The air holes are arranged in a square shape, and the air holes on both sides of the central air hole are missing, forming two cores A and B to transmit light, as shown in Figure 1a. In Figure 1a, Λ = 3.5 μm, d = 2.8 μm, and d_c_ = 2.8 μm represent the pitch of the air holes, and diameter of the air hole, the diameter of the central air hole, respectively. To excite SPR, a metal thin film of thickness h is plated around the central air hole. The cladding material is quartz glass, with a refractive index n = 1.45. The refractive index of the central air hole is represented by n_a_, and different functional materials are filled in the air hole in order to realize the sensing of different functions. Figure 1b shows that the schematic diagram of the proposed PCF-SPR sensor in the three-dimensional (3D) model. The existing technology can already manufacture PCFs. The PCFs can be produced by the stake-and-draw method. The preform of proposed PCF can be made of solid rods, capillaries, and thicker wall capillaries through stacking, then, the preform is drawn into semi-finished PCF using the drawing device [24]. The metal is deposited by chemical vapor deposition or sputtering. When considering that magnetic field sensors have important application values in the fields of geophysical surveying and navigation, military equipment applications, biomedical sensing, and aerospace field [25,26,27]. In this article, we chose to add a magnetic fluid to make a photonic crystal fiber magnetic field sensor and study its magnetic field sensing characteristics. Figure 1c is a schematic diagram of the experimental setup of the proposed sensor. Using a broadband light source as the incident light, pass through a polarization controller, and enter the designed PCF-SPR sensor. We can use the magnetic coil to generate the magnetic field and use the Gauss meter to calibrate the magnetic field. Finally, the output light is detected by an optical spectrum analyzer (OSA).

**Figure 2:**
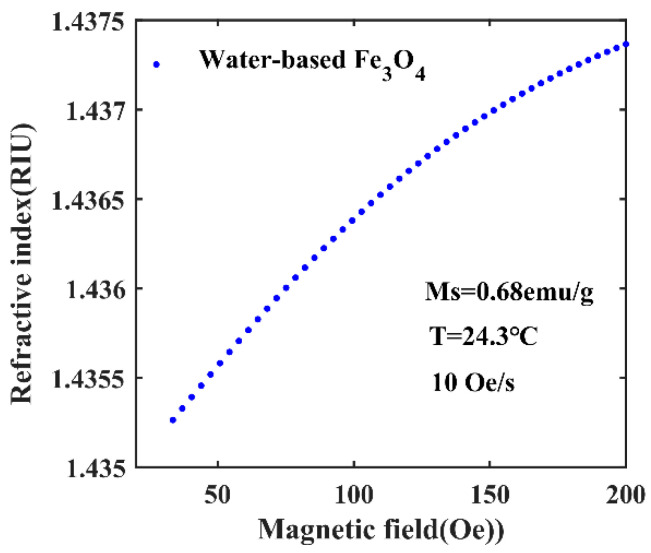
Variation of refractive index of magnetic fluid with magnetic field.

In the simulation, a metal layer is plated around the core and the evanescent field of light is used to excite SPR. The dielectric constant of the metal layer is expressed by Drude model, as follows [28]:

(1) $$\varepsilon (\omega )=\varepsilon_{\infty }-\frac{\omega_{p}^{2}}{\omega (\omega +i\omega_{c})}$$

where *ω_c_* is the collision frequency, *ω_p_* is the plasma frequency, and *ε_∞_* isassociated with the absorption peaks at high frequency (*ω* >> *ω_c_*). These parameters fit well into the experimental data in the literature [29]. The change of the refractive index of the magnetic fluid with the magnetic field can be expressed by the following function [30]:

(2) $$n_{\mathrm{mf}}(H,T)=[n_{s}-n_{0}]\left[\coth\left(\alpha \frac{H-H_{c,n}}{T}\right)-\frac{T}{\alpha \left(H-H_{c,n}\right)}\right]+n_{0}$$

where *n*_mf_ is the refractive index of the magnetic fluid, *n_s_* is the saturation value of the refractive index, *n*_0_ is the initial refractive index, *α* is the fitting coefficient, *T* is the working emperature, and *H_c,n_* is the threshold. When the external magnetic field *H* is greater than *H_c,n_*, according to formula (2), it can be calculated that *n*_mf_ is a constant. By adjusting the concentration of the magnetic fluid, the refractive index of the magnetic fluid can be controlled [30]. At the same time, temperature and particle size also affect the refractive index of magnetic fluids [31]. The type of magnetic fluid used is water-based Fe_3_O_4_ magnetic fluid. The initial parameters were set to *T* = 24.3 °C, *α* = 5, *H_c,n_* = 30 Oe, *n*_0_ = 1.4352, *n_s_* = 1.4385, magnetic fluid concentration = 0.68 emu/g, and the film thickness is 11.8 μm, working temperature is 24.3 °C, and sweep rate of the field is 10 Oe/s [32]. Figure 2 shows the curve of the refractive index of water-based Fe_3_O_4_ magnetic fluid with magnetic field.

The loss spectrum determines the sensing performance of the PCF-SPR sensor. By analyzing the loss spectrum under different conditions, the spectral sensitivity and detection accuracy of the sensor can be further calculated and discussed [33]. The confinement loss is the main factor affecting the loss spectrum, which can be calculated by the imaginary part of the effective refractive index of the mode. The formula is as follows [34]:

(3) αloss=8.686×2πλIm[neff]×107(dB/cm)

*λ* is the wavelength of incident light in vacuum and Im[n_eff_] is the imaginary part of the effective refractive index of the fiber core mode. The equation shows that the optical fiber transmission loss is proportional to the imaginary part of the effective refractive index. When the refractive index changes, the phase matching wavelength between the fiber core mode and the SPP mode changes. Correspondingly, the resonance peak in the loss spectrum changes with the change of the phase matching wavelength, and the sensing can be realized by tracking the wavelength change of the resonance peak [13].

**Figure 3:**
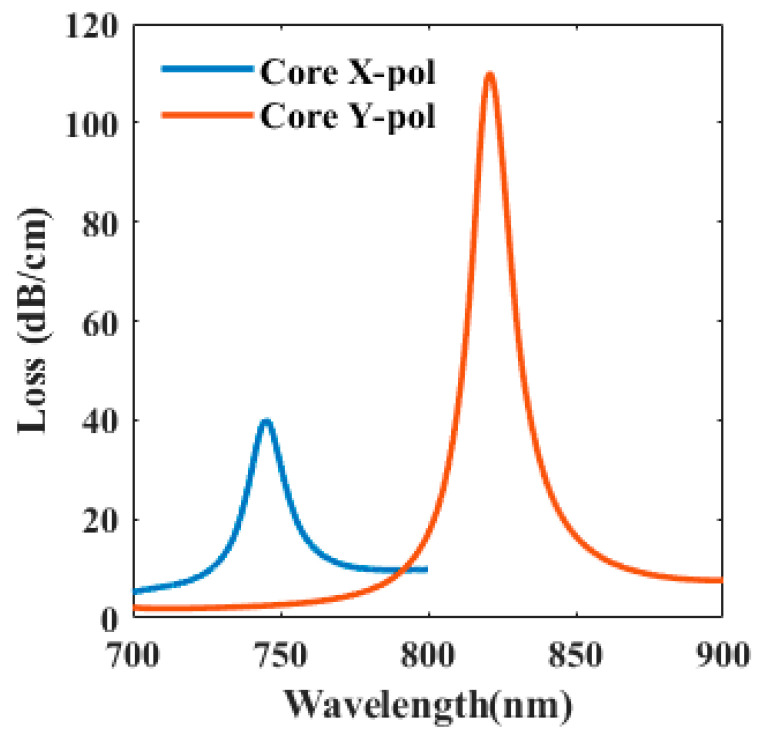
Comparison of the loss spectra of x-polarized fiber core mode and the loss spectra of y-polarized fiber core mode.

We used COMSOL Multiphysics 5.4 based on finite element analysis in order to simulate the proposed sensor structure. In order to approach the ideal situation, a perfect matching layer (PML) is added to the outermost area to absorb energy. The mesh generates a free triangle mesh. The complete mesh contains 16,502 domain elements and 1338 boundary elements. The number of degrees of freedom solved is 108,709. Both the x-polarized fiber core mode and the y-polarized fiber core mode can excite SPR due to the characteristics of the dual-core photonic crystal fiber. The Figure 3 shows that comparison of the loss spectra of x-polarized fiber core mode and the loss spectra of y-polarized fiber core mode. By comparison, it is found that under the same conditions, the polarization mode in the x-polarized is obviously not as good as the y-polarized in the excitation of SPR. When considering that our sensors need to use SPR for sensing, we choose y-polarized fiber core mode for study. 

**Figure 4:**
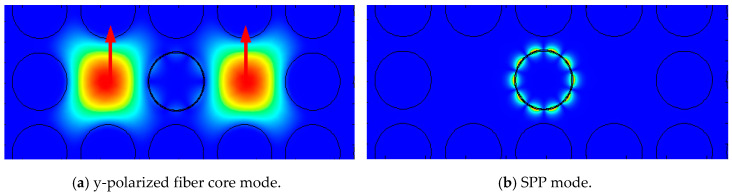
The optical field distribution of (**a**) the y-polarized fiber core mode and (**b**) SPP mode and the arrows represent the direction of the electric field.

For a given wavelength and central air hole refractive index n_a_, the model analysis results are shown in Figure 4. As Figure 4a,b show, the optical field distribution of the y-polarized fiber core mode and SPP mode and the arrows represent the direction of the electric field. This shows that, in a certain wavelength range, when the phase matching is satisfied, the energy in the PCF is transferred from the y-polarized fiber core mode to the SPP mode, and the energy loss is mainly caused by the excitation of the SPP mode. The SPP mode is highly lossy, and the loss in this wavelength range increases significantly. The resonance wavelength is the wavelength at which the loss peak in the loss spectrum is the largest [6]. Theoretically, when the resonance wavelength appears, the propagation constants of the two modes are required to be equal, which means that the effective refractive index of the two modes must be close. Therefore, when the refractive index of the central air hole changes, the resonance wavelength will also change. The principle of the PCF-SPR magnetic field sensor based on surface plasmon resonance is that SPR has extremely high sensitivity to changes in the refractive index of the surrounding medium and PCF’s special air hole structure. When PCF air holes are filled with different functional material, it can realize different sensing functions. Add magnetic fluid to the central air hole, when the external magnetic field changes, the refractive index of the magnetic fluid will change, the resonance wavelength will also change. The sensitivity of the PCF-SPR magnetic field sensor can be obtained by tracking the change of the resonance wavelength of the loss spectrum.

## 3. Simulations and Analysis

**Figure 5:**
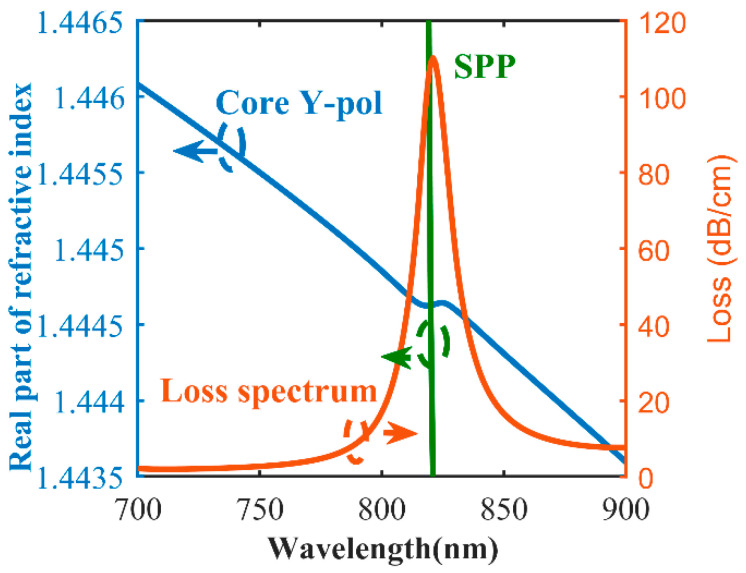
Real part of effective refractive index of the y-polarized fiber core mode and surface plasmon polariton (SPP) mode, and the loss spectra of the PCF-SPR sensor when the material of the metal layer is gold.

**Figure 6:**
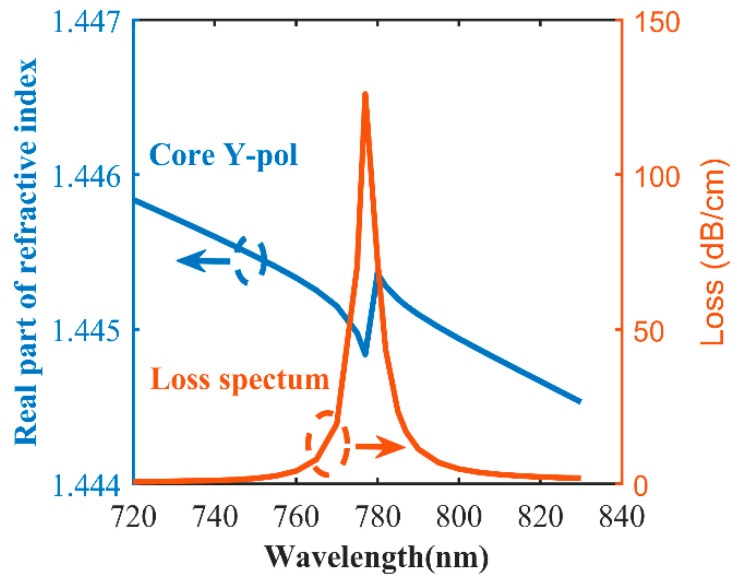
Real part of effective refractive index of the y-polarized fiber core mode, and the loss spectra of the PCF-SPR sensor when the material of the metal layer is silver.

Figure 5 shows the real part of the effective refractive index of the y-polarized fiber core mode and SPP mode and the loss spectrum of the y-polarized fiber core mode when n_a_ = 1.44 and the thickness of the gold layer is 50 nm. The blue line is the real part of the effective refractive index of the y-polarized fiber core mode, the green line is the real part of the effective refractive index of the SPP mode, and the red line is the loss spectrum of the y-polarized fiber core mode. It can be seen that the dispersion relationship between the y-polarized fiber mode core and the SPP mode has an obvious intersection, as shown in Figure 5. As the wavelength increases, the loss of the y-polarized fiber mode first increases and then decreases. When the real refractive index of the y-polarized fiber core mode is equal to the real refractive index of the SPP mode, which is, the phase matching condition is satisfied, the loss of y-polarized fiber core mode reaches its maximum at 821 nm. Obviously, high coupling occurs at the wavelength of 821 nm, and the energy loss of the y-polarized fiber core mode is mainly caused by the SPP mode that is generated by excitation, and the loss of y-polarized fiber core mode is the largest at the wavelength of 821 nm. The blue line of effective refractive index representing the y-polarized fiber core mode has an s-shaped kink at the resonance wavelength as it drops. The reason for the s-shaped kink is that, when resonance occurs, free electrons cause resonant electrons to oscillate, which affects the evanescent field, resulting in a change in the mode of the PCF, and then the effective refractive index of PCF changes suddenly. The stronger the resonance, the more obvious the s-shaped kink. Figure 6 shows the real part of the effective refractive index and the loss spectrum of the y-polarized fiber core mode when n_a_ = 1.44 and the thickness of the silver layer is 50 nm. The blue line is the real part of the effective refractive index of the y-polarized fiber core mode, and the red line is the loss spectrum of the y-polarized fiber core mode. As the wavelength increases, the loss of the y-polarized fiber mode first increases and then decreases. When the phase matching condition is satisfied, the loss of y-polarized fiber core mode reaches its maximum at 777 nm. When comparing with Figure 5, it can be found that when silver is used as the coating material, the loss peak is sharper, and the s-shaped kink is more obvious. This is because the nature of silver is more active.

**Figure 7:**
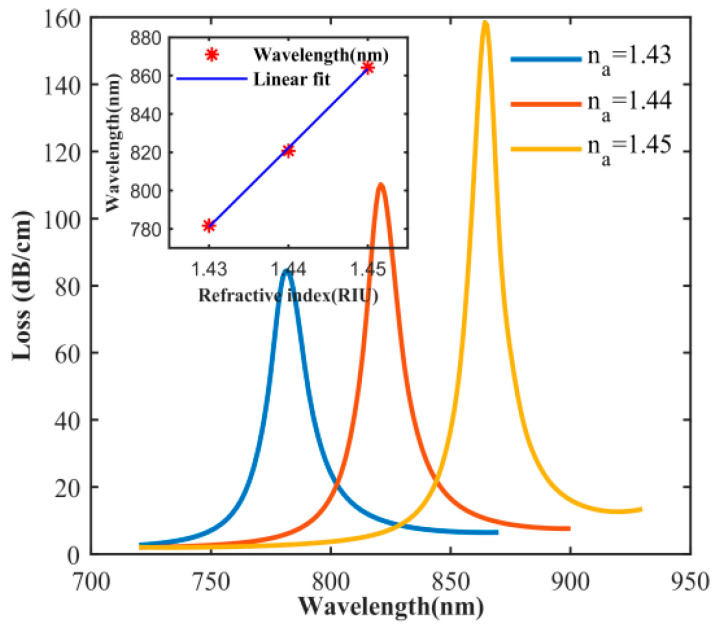
Influence of n_a_ on the loss spectrum for a gold coating thickness of 50 nm (insert Figure 7 Variations of resonance wavelength with different RIs (n_a_) from 1.43 to 1.45).

When the refractive index of the central air hole changes, the phase matching condition and the resonance wavelength also change. Figure 7 shows the effect of different central pore refractive index on the designed sensor, which, under the condition that other parameters remain unchanged, the thickness of the gold layer is 50 nm, the loss spectrum when n_a_ is 1.43, 1.44, and 1.45, respectively. In Figure 7, the peak losses at the resonance wavelengths of 782 nm (n_a_ = 1.43), 821 nm (n_a_ = 1.44) and 864 nm (n_a_ = 1.45) are 84.5 dB/cm, 110.1 dB/cm and 158.4 dB/cm, respectively. Obviously, as n_a_ increases, the resonance wavelength is red-shifted, and the peak loss increases linearly. The main reason is that with the continuous increase of n_a_, the difference between the overall refractive index of the photonic crystal fiber and the refractive index of the central air hole increases, and the excited SPP mode is enhanced. Sensitivity is an important parameter of the sensor, which describes the change of the resonant wavelength of the sensor to the unit refractive index, which can be defined, as [35]:

(4) $$S_{\lambda }=\frac{\Delta \lambda_{\mathrm{peak}}}{\Delta n_{a}}(\mathrm{nm}/\mathrm{RIU})$$

where Δ*λ*_peak_ is the distance of the loss peak drift and Δn_a_ is the change value of the refractive index of the central air hole. The inset in Figure 7 shows the change in the resonant wavelength of the refractive index of n_a_ between 1.43 and 1.45. The red dot represents the resonance wavelength and the blue line is a linear fit. We can see that there is a linear relationship between the refractive index and the resonance wavelength. The results show that the sensor has good linear response. In the inset of Figure 7, the slope is the sensitivity and the sensitivity of the sensor is 4125 nm/RIU.

**Figure 8:**
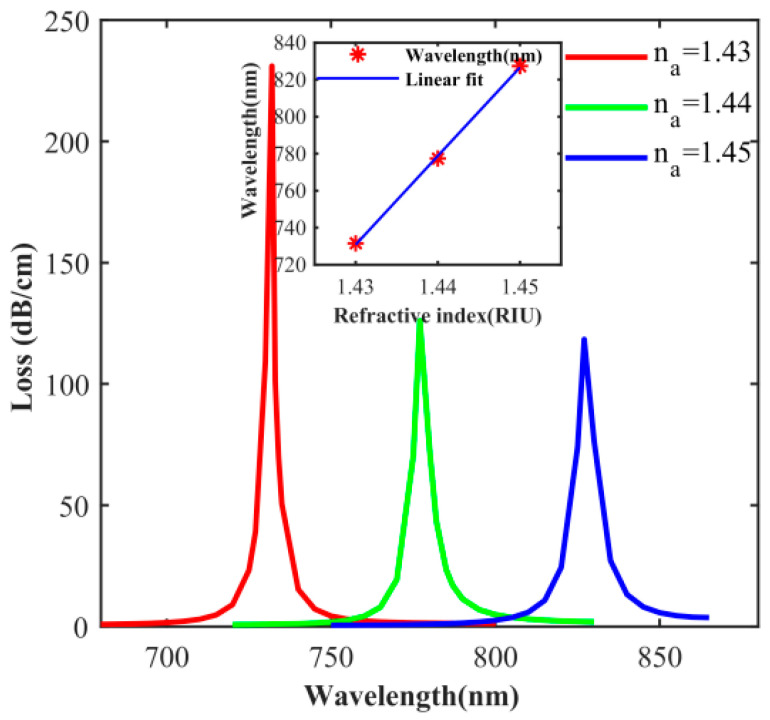
Influence of n_a_ on the loss spectrum for a silver coating thickness of 50 nm (insert Figure 8 Variations of resonance wavelength with different RI (n_a_) from 1.43 to 1.45).

The use of different coating materials will affect the performance of the designed sensor. Figure 8 shows that, when the condition that other parameters remain unchanged, the thickness of the silver layer is 50 nm, the loss spectrum when n_a_ is 1.43, 1.44, and 1.45 respectively. In Figure 8, as the refractive index increases, the resonant wavelength red shifts and the loss decreases. The peak losses at the resonance wavelengths of 732 nm (n_a_ = 1.43), 777 nm (n_a_ = 1.44) and 827 nm (n_a_ = 1.45) are 231 dB/cm, 127 dB/cm, and 118.4 dB/cm, respectively. When silver is used as the material, the loss is obviously larger than that of gold. At the same time, with the continuous increase of n_a_, the peak loss is obviously reduced but not linearly. The reason is that the dielectric constant of the silver has changed. The inset in Figure 8 shows the change in the resonant wavelength of the refractive index of n_a_ between 1.43 and 1.45. In the inset of Figure 8, the slope is the sensitivity and the sensitivity of the sensor is 4755 nm/RIU. A comparison of the sensitivity of the proposed PCF-SPR when the material of the metal layer are Au and silver is shown in Table 1.

Figure 7 and Figure 8 compare that, when silver is used as a material, the sensor sensitivity is slightly higher than gold, the loss peak is sharper, and the resolution will be higher. The reason is that the nature of silver itself is more active than gold, and the free electrons are more active. However, at the same time, it can be found that when gold is used as the material, the linearity and stability of the sensor are stronger than that of silver. Gold will be more suitable for magnetic field sensors when considering that the magnetic fluid is corrosive and the sensitivity is not greatly improved.

**Figure 9:**
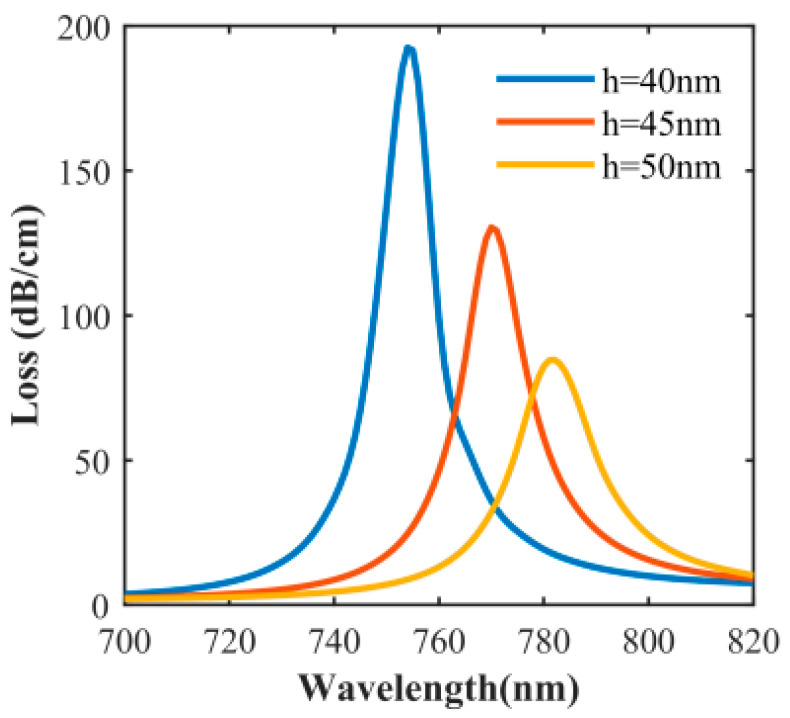
Influence of h_Au_ on the loss spectrum with n_a_ = 1.43.

Surface plasmon resonance is very sensitive to the thickness of the metal layer. The thickness of the gold layer is also an important factor affecting the half-width and amplitude of the resonance peak, so the influence of the thickness of gold layer on the sensor should also be considered. Figure 9 shows that the influence of the thickness of the gold layer on the sensor performance under n_a_ = 1.43 when other parameters are unchanged. As shown in Figure 9, the resonance wavelength is red-shifted and the loss peak value decreases with the increase of the thickness of gold layer. In Figure 9, under the condition of n_a_ = 1.43, the thickness of the gold layer is 40 nm, the peak loss is 192.5 dB/cm, and the resonance wavelength is 754 nm; when the thickness of the gold layer is 45 nm, the peak loss is 130.4 dB/cm, and the resonance wave moves to 770 nm; when the thickness of the gold layer is 50 nm, the peak loss is 84.7 dB/cm, and the resonance wavelength is 782 nm. Obviously, when the thickness of the gold layer increases from 40 nm to 50 nm, the resonance wavelength shifts to a longer wavelength, and the peak loss decreases sharply. The reason is that the thickness of the gold layer increases from 40 nm to 50 nm. When the metal thickness becomes thicker, the central air hole diameter is unchanged, the metal surface is closer to the core mode field, so that the contact area between the core mode field and the metal surface increases, and more light is coupled to the metal surface, and the resonance wavelength is red-shifted [36]. However, at the same time, the SPP mode decays quickly in the vertical direction. When the thickness of the gold film increases, the SPPs mode strength is weakened and the loss sharply decreases. 

**Figure 10:**
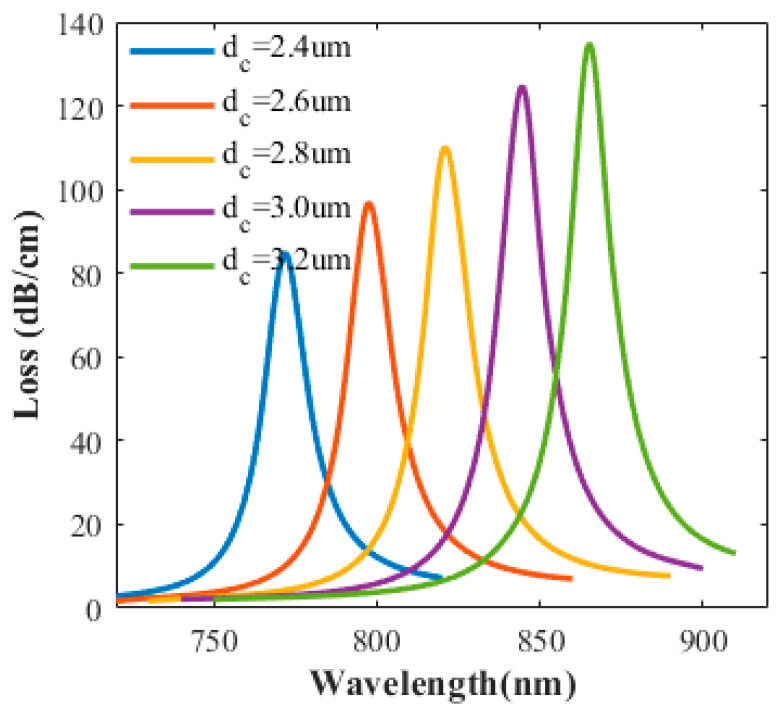
Influence of d_c_ on the loss spectrum with h_Au_ = 50 nm.

The diameter of the central air hole is also one of the important factors affecting sensor parameters. Figure 10 shows the Influence of d_c_ on the loss spectrum under h_Au_ = 50 nm when other parameters are unchanged. As shown in Figure 10, when the thickness of the gold layer does not change, as the diameter of the central pore increases, the loss increases, and the loss peak is red-shifted. This is because that, when the metal thickness becomes thicker, the central air hole diameter is unchanged, the metal surface is closer to the core mode field, so that the contact area between the core mode field and the metal surface increases, and more light is coupled to the metal surface, the loss increases. The reason is that the increase in diameter will make the core mode closer to the metal, and the coupling effect will be stronger, the excited SPP mode is more enhanced. The thickness of the gold layer and the diameter of the central air hole have great influence on the PCF-SPR sensor. When the gold layer is too thick and the diameter of the central air hole is too small, the loss will decrease and affect the peak value; when the gold film is too thin, the energy of the SPP mode at the film will be weak, which is unfavorable for SPR excitation, but the increase in diameter will make the core mode closer to the metal and the excited SPP mode is enhanced. Therefore, the choice of thickness and the diameter of the central air hole should consider the peak position and height as well as SPP excitation.

**Figure 11:**
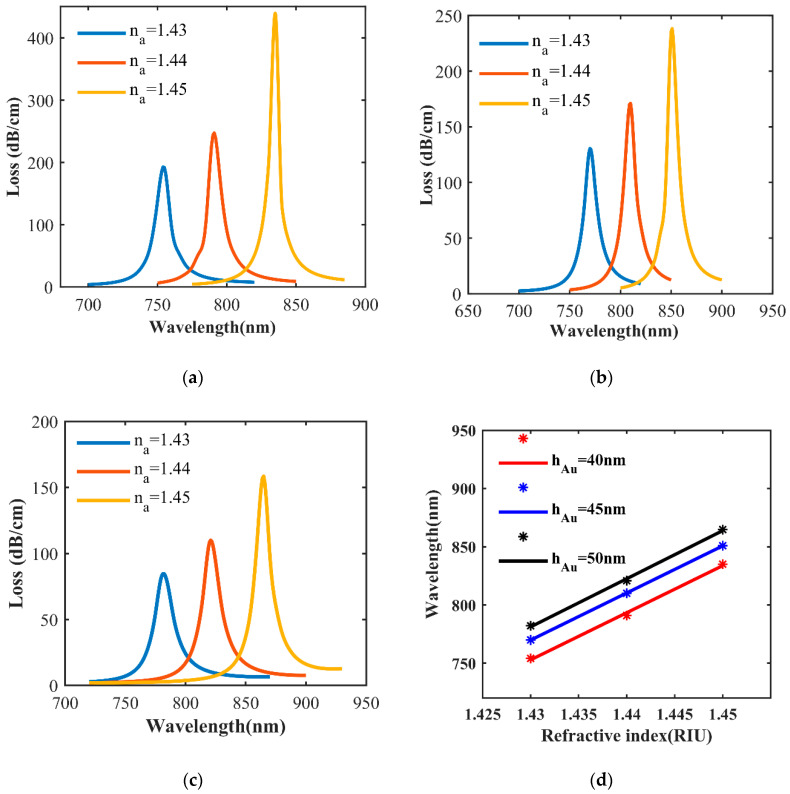
Influence of RI on the loss spectrum with different h_Au_ from 40 nm to 50 nm. (**a**) h_Au_ = 40 nm, (**b**) h_Au_ = 45 nm, (**c**) h_Au_ = 50 nm, and (**d**) the fitted results of resonant wavelength of y-polarized fiber core mode with different h_Au_, where the red, blue, and black markers are the simulation results.

In summary, the thickness of the metal film, the metal material, and the refractive index of the central air hole affect the resonance wavelength and loss peak. Therefore, Figure 11a–c describe the loss spectra of n_a_ = 1.43, 1.44, and 1.45 when the h_Au_ is 40 nm, 45 nm, and 50 nm, respectively, when gold is selected as the coating material. As shown in Figure 11a, when h_Au_ = 40 nm, n_a_ = 1.43, peak loss is 192.5 dB/cm, resonance wavelength is 754 nm; n_a_ = 1.44, peak loss is 247.2 dB/cm, resonance wavelength is 791 nm; and, n_a_ = 1.45, peak loss is 439.4 dB/cm, resonance wavelength is 835 nm. The loss peak is gradually red-shifted. The main reason for this phenomenon is that the change of n_a_ causes the effective refractive index of the SPP mode to change, which causes the position of the intersection of the y-polarized fiber core mode and the SPP mode to move, and then causes the loss peak to move. At the same time, the overall effective refractive index of the sensor increases as the n_a_ increases, resulting in an increase in coupling efficiency, and the energy transmitted to the metal increases, the loss caused by SPP increases. The relationship between the resonance wavelength and n_a_ under different h_Au_ is shown in Figure 11d. The red, blue, and black marks are the simulation results when h_Au_ is 40 nm, 45 nm, and 50 nm, respectively, and the red, blue, and black lines represent the resonance wavelength fitting lines when h_Au_ is 40 nm, 45 nm, and 50 nm, respectively. In Figure 11d, the fitted lines of the red, blue, and black lines are linear, which means that the designed sensor has good linearity. Table 2 shows the refractive index sensitivity calculated according to Formula (4).

**Figure 12:**
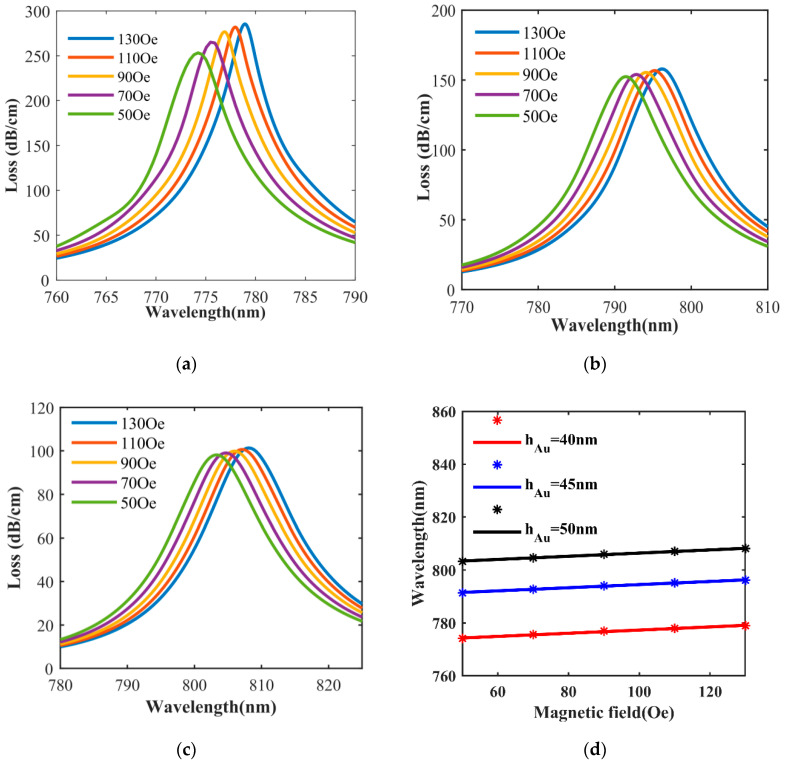
Influence of magnetic field intensity on the loss spectrum with different h_Au_ from 40 nm to 50 nm, (**a**) h_Au_ = 40 nm, (**b**) h_Au_ = 45 nm, (**c**) h_Au_ = 50 nm, and (**d**) the fitted results of resonant wavelength of y-polarized fiber core mode with different magnetic field intensity, where the red, blue, and black markers are the simulation results.

Based on the above discussion, we have studied the magnetic field sensitivity of the PCF-SPR sensor between 50–130 Oe. The water-based Fe_3_O_4_ magnetic fluids added to the central air hole of the sensor, when the external magnetic field changes, the refractive index of the magnetic fluid changes, the resonance wavelength changes, and the y-polarized fiber core mode loss spectrum drifts. The magnetic field sensitivity of the sensor can be obtained by calculating the drift of the loss spectrum. Magnetic field sensitivity is defined as:

(5) $$S_{H}=\frac{\Delta \lambda_{\mathrm{peak}}}{\Delta H}(\mathrm{nm}/Oe)$$

where, Δ*λ*_peak_ is the distance of the loss peak drift and Δ*H* is the change of the magnetic field. Figure 12a–c describe, when the magnetic field strength is in the range of 50–130 Oe, the loss spectrum of the central air hole when the thickness of gold layer is h_Au_ equal to 40 nm, 45 nm, and 50 nm, respectively. It can be found that, at the same metal thickness, the loss peak resonance wavelength increases with the increase of the magnetic field, and the peak loss increases. The reason is that, according to Formula (2), as the magnetic field increases, the refractive index of the magnetic fluid in the central air hole becomes larger, and the overall effective refractive index of the sensor component increases with the increase in n_a_, resulting in the coupling efficiency being enhanced, and the energy transmitted to the metal increasing, the loss peak red-shifting, and the loss increasing significantly. The relationship between the resonance wavelength and magnetic field under different h_Au_ is shown in Figure 12d. The red, blue, and black marks are the simulation results when h_Au_ is 40 nm, 45 nm, and 50 nm, respectively, and the red, blue, and black lines represent the resonance wavelength fitting lines when h_Au_ is 40 nm, 45 nm, and 50 nm, respectively. Table 3 shows the magnetic field sensitivity calculated according to Formula (5).

According to the simulation results, it can be found that the sensing performance of the sensor is related to the coating material, the refractive index, and diameter of the central air hole and the coating thickness. The choice of coating material needs to balance sensitivity and practicability. The refractive index of the central air hole affects the loss peak drift degree of the sensor, and the thickness of the metal coating determines loss peak, but the diameter will affect mode coupling, thus affecting the excitation of SPP mode. Therefore, we can choose different sensor parameters to achieve the desired performance. When the central air hole is filled with magnetic fluid, the magnetic fluid is corrosive, and gold as the coating material is more suitable for magnetic field sensors. At the same time, we fitted the sensor resonance wavelength and found that the designed sensor has good linearity in refractive index range from 1.43–1.45. We also analyzed and discuss the influence of the thickness of the gold layer and the diameter of the center air hole. When we choose sensor parameters, we should consider the peak position and height and SPP excitation.

## 4. Conclusions

In summary, a new type of PCF-SPR magnetic field sensor is designed and analyzed while using finite element method. In the article, we study the mode field characteristics of the proposed sensor to prove its sensing characteristics. Subsequently, the sensor parameters were changed, and the effects of metal materials, metal thickness, the diameter of the central air hole, and refractive index of the central air hole on the sensing performance were discussed. The simulation results show that the sensor is very sensitive to the change of refractive index and has good linearity in refractive index range from 1.43–1.45. The thickness and the diameter of the central air hole will affect the peak position and height and SPP excitation. When the thickness of gold layer is 50 nm, d_c_ = 2.8 μm, the refractive index sensitivity is 4125 nm/RIU, and the thickness of silver layer is 50 nm, d_c_ = 2.8 μm, the refractive index sensitivity is 4755 nm/RIU in refractive index range from 1.43–1.45. When the designed sensor is used for magnetic field sensing, the loss spectrum is red-shifted with the increase of the magnetic field. By optimizing the sensor structure parameters, the magnetic field sensitivity and resolution of the sensor can be improved. The highest magnetic field sensitivity can reach 61.25 pm/Oe in the range from 50 Oe to 130 Oe. The sensor not only has high sensitivity of refractive index, but also can realize accurate measurement of magnetic field. It has great potential in complex environment, remote sensing, real-time monitoring, and other fields, and it has potential application prospects for the development of high-resolution magnetic field sensors. At the same time, the function of the sensor can be changed with the change of the filling material of the central air hole due to the special structure of the PCF. For example, it can be filled with a temperature-sensitive liquid to make a temperature sensor, or it can be filled with a liquid with a greater change in refractive index in order to improve the sensitivity of the sensor which will be researched in the future.

## Figures and Tables

**Table 1:** Comparison of the refractive index sensitivity of the proposed PCF-SPR when the material of the metal layer are Au and silver.

Material	S (nm/RIU)
Au	4125
Ag	4755

**Table 2:** Comparison of the refractive index sensitivity of the proposed PCF-SPR with different h_Au_ from 40 nm to 50 nm.

h_Au_	S (nm/RIU)
40 nm	4050
45 nm	4055
50 nm	4125

**Table 3:** Comparison of the magnetic field sensitivity of the proposed PCF-SPR with different h_Au_ from 40 nm to 50 nm.

h_Au_	S (nm/Oe)
40 nm	57.5
45 nm	58.75
50 nm	61.25
